# Supplementary material for: Deep learning and single-cell phenotyping for rapid antimicrobial susceptibility detection in Escherichia coli
Source: Commun Biol. 2023 Nov 14;6:1164. doi: 10.1038/s42003-023-05524-4 (PMC10645916; doi:10.1038/s42003-023-05524-4)
Supplement: Supplementary file 2 — SUPPLEMENTAL MATERIAL [file 42003_2023_5524_MOESM2_ESM.pdf]

Deep Learning and Single-Cell Phenotyping for Rapid Antimicrobial Susceptibility Detection in Escherichia coli

Supplementary Information

Alexander Zagajewski, Piers Turner, Conor Feehily, Hafez El Sayyed, Monique Andersson, Lucinda Barrett, Sarah Oakley, Mathew Stracy, Derrick Crook, Christoffer Nellåker, Nicole Stoesser & Achillefs N. Kapanidis.

Antibiotic	Code	EUCAST breakpoint (mg/L)	Treatment concentration (mg/L)	Treatment duration (min)
Ciprofloxacin	CIP	0.5	10	30
Gentamicin	GENT	2	40	30
Rifampicin	RIF	-	100	30
Co-amoxiclav	COAMOX	8/2	120/60	60

Table S1. Treatment conditions and abbreviations used to generate susceptible phenotype training data in MG1655 *E. coli*. Antibiotic column contains the full, standard name of the antibiotic. The code refers to the abbreviated names for these antibiotics used in this publication. The EUCAST breakpoints are the standardised thresholds used clinically for classifying specimens as either resistant or susceptible, via growth inhibition on agar plate, in our setting. No breakpoint has been established for the use of rifampicin in *E. coli*. The treatment concentration and duration are the antibiotic exposure parameters used to generate susceptible phenotypes in this study.

ABX	TEST	TP	TN	FP	FN	Precision	Recall	Accuracy
CIP	CROSS VALIDATION	2059	2653	197	143	91%	94%	93%
	HOLDOUT	422	467	33	78	93%	84%	89%
GENT	CROSS VALIDATION	1629	714	160	57	91%	97%	92%
	HOLDOUT	468	459	41	32	92%	94%	93%
RIF	CROSS VALIDATION	3808	2454	396	466	91%	89%	88%
	HOLDOUT	402	457	43	98	90%	80%	86%
COAMOX	CROSS VALIDATION	957	622	252	210	79%	82%	77%
	HOLDOUT	352	322	78	48	82%	88%	84%

Table S2. Cross-validation and holdout detection results in the *E. coli* MG1655 training strain across all antibiotics used in the study. The cross-validation and holdout test (holdout) experiments were carried out on 4 different antibiotics (abx) used in the study. True positive (TP) is the number of treated cells correctly classified as susceptible. True negative (TN) is the number of untreated cells correctly classified in the resistant class (showing no response due to being untreated). False positive (FP) and false negative (FN) are the numbers of misclassified untreated cells and treated cells respectively. From these counts, the precision, recall and accuracy can be calculated.

ABX	Fold	TP	TN	FP	FN	Precision	Recall	Accuracy
CIP	1	477	464	23	36	95%	93%	94%
	2	458	397	42	103	92%	82%	86%
	3	404	494	96	6	81%	99%	90%
	4	442	487	58	13	88%	97%	93%
	5	473	413	27	87	95%	84%	89%
	6	393	484	107	16	79%	96%	88%
	**Average**	**2647**	**2739**	**353**	**261**	**88%**	**91%**	**90%**
GENT	1	469	346	31	154	94%	75%	82%
	2	490	422	10	78	98%	86%	91%
	3	467	422	33	78	93%	86%	89%
	4	147	479	353	21	29%	88%	63%
	5	391	476	109	24	78%	94%	87%
	6	456	482	44	18	91%	96%	94%
	**Average**	**2420**	**2627**	**580**	**373**	**81%**	**87%**	**84%**
RIF	1	475	325	25	175	95%	73%	80%
	2	365	462	135	38	73%	91%	83%
	3	441	458	59	42	88%	91%	90%
	4	465	445	35	55	93%	89%	91%
	5	340	352	160	148	68%	70%	69%
	6	396	397	104	103	79%	79%	79%
	**Average**	**2482**	**2439**	**518**	**561**	**83%**	**82%**	**82%**
COAMOX	1	312	216	88	184	78%	63%	66%
	2	297	88	103	12	74%	96%	77%
	3	258	272	142	128	65%	67%	66%
	4	316	275	84	125	79%	72%	74%
	5	297	219	103	181	74%	62%	65%
	6	300	252	100	148	75%	67%	69%
	**Average**	**1780**	**1322**	**620**	**778**	**74%**	**70%**	**69%**

Table S3. K-fold cross-validation detection results in the *E. coli* MG1655 training strain across all antibiotics used in the study. The K-fold cross-validation experiments were carried out on 4 different antibiotics (abx) used in the study. True positive (TP) is the number of treated cells correctly classified as susceptible. True negative (TN) is the number of untreated cells correctly classified in the resistant class (showing no response due to being untreated). False positive (FP) and false negative (FN) are the numbers of misclassified untreated cells and treated cells respectively. From these counts, the precision, recall and accuracy can be calculated. The statistics for each fold and the average of all folds in each cross validation experiment are shown.

**Isolate name**	**Genotype**	**MIC (mg/L)**
EC1	*marR*^{N3}	0.008
EC2	*marR*^{N3}	0.03
EC3	*gyrA*^{L83} *parC*^{I80}	0.5
EC4	*gyrA*$^{L83, N87}$ *parC*^{I80}	8
EC5	*gyrA*$^{L83, N87}$ *parC*$^{I80, V84}$ *parE*^{L529}	72
EC6	*gyrA*$^{L83, N87}$ *parC*^{I80} *parE*^{A458}	108

Table S4. Table of clinical E. coli isolates used in the study. Isolate name refers to the pseudorandom code used for isolates in the manuscript and figures. Genotype contains details of mutations associated with resistance to ciprofloxacin, detected by whole genome sequencing of the isolates, following the method described in Methods. The MIC column lists experimentally derived ciprofloxacin Minimum Inhibitory Concentrations (MIC), following the method described in Methods.

Clinical Isolate	MIC (mg/L)	Treatment Concentration (mg/L)	Treated Sensitive Classification Ratio	Pearsons R	Sample Size
EC1	0.008	10	0.72	0.54	105
EC2	0.03	10	0.86	0.71	61
EC3	0.5	10	0.80	0.72	60
EC4	8	10	0.28	0.29	356
EC5	72	10	0.03	0.05	14203
EC6	108	10	0.25	0.05	14204

Table S5. The number of observations (sample size) required to classify treated susceptible phenotypes with a 90 % accuracy and 1% statistical significance, calculated from the data in Figure 5. Pearson's R was used as the effect size.

Model	Classification Accuracy (%)					
	COAMOX	GENT	CIP	RIF	Mean	Std.Dev
DenseNet 121	82%	91%	89%	85%	87%	4%
DenseNet 201	75%	94%	71%	81%	80%	9%
EfficientNet b0	78%	89%	66%	73%	76%	8%
EfficientNet b7	68%	57%	59%	56%	60%	5%
Mask R-CNN	54%	83%	78%	68%	71%	11%
ResNet 18	78%	92%	91%	83%	86%	6%
ResNet 50	77%	96%	85%	76%	83%	8%
ResNet 101	72%	89%	85%	69%	79%	8%
ResNet 152	74%	86%	81%	62%	76%	9%
VGG 11	70%	80%	59%	52%	65%	11%
VGG 19	70%	85%	59%	52%	67%	12%
YOLO v8	47%	97%	69%	99.8%	78%	22%

Table S6. Table of classification accuracies for a range of different convolutional neural network architectures, evaluated for four antibiotic phenotypes on the cross-validation test set. The mean and standard deviation are shown for each classification model.

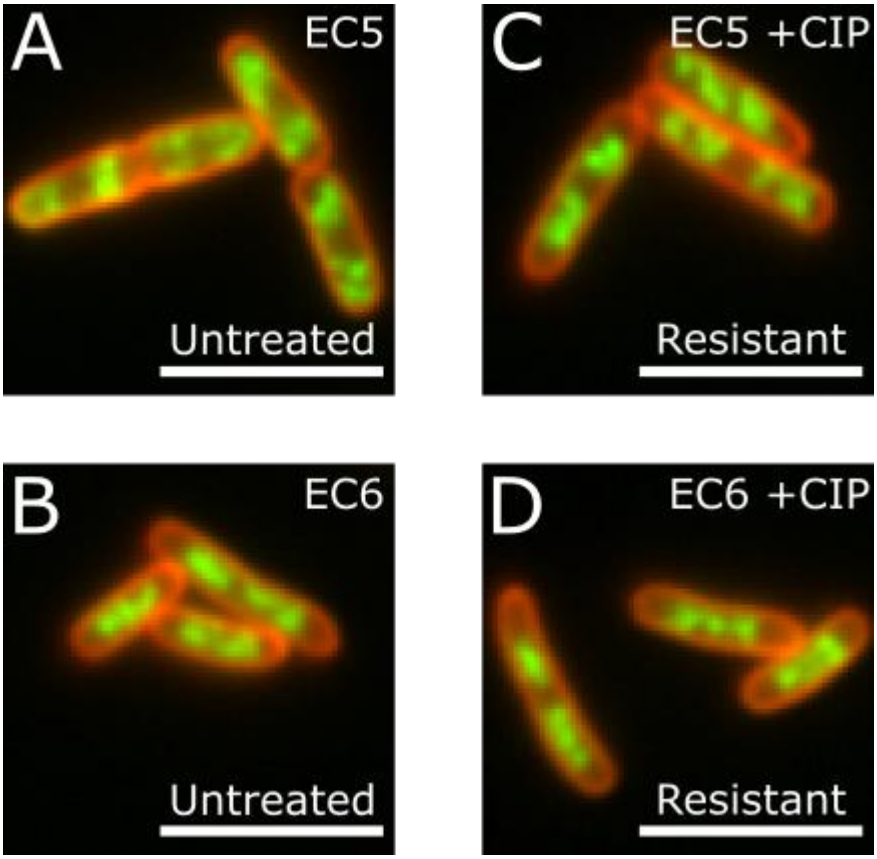

Fig S1. Comparison of the resistant and untreated phenotype in ciprofloxacin resistant clinical isolates. (A-B) Untreated phenotype in ciprofloxacin resistant EC5 and EC6. (C-D) Resistant phenotype in EC5 and EC6, after treatment with ciprofloxacin. The scalebars in (A-D) are 2 µm.

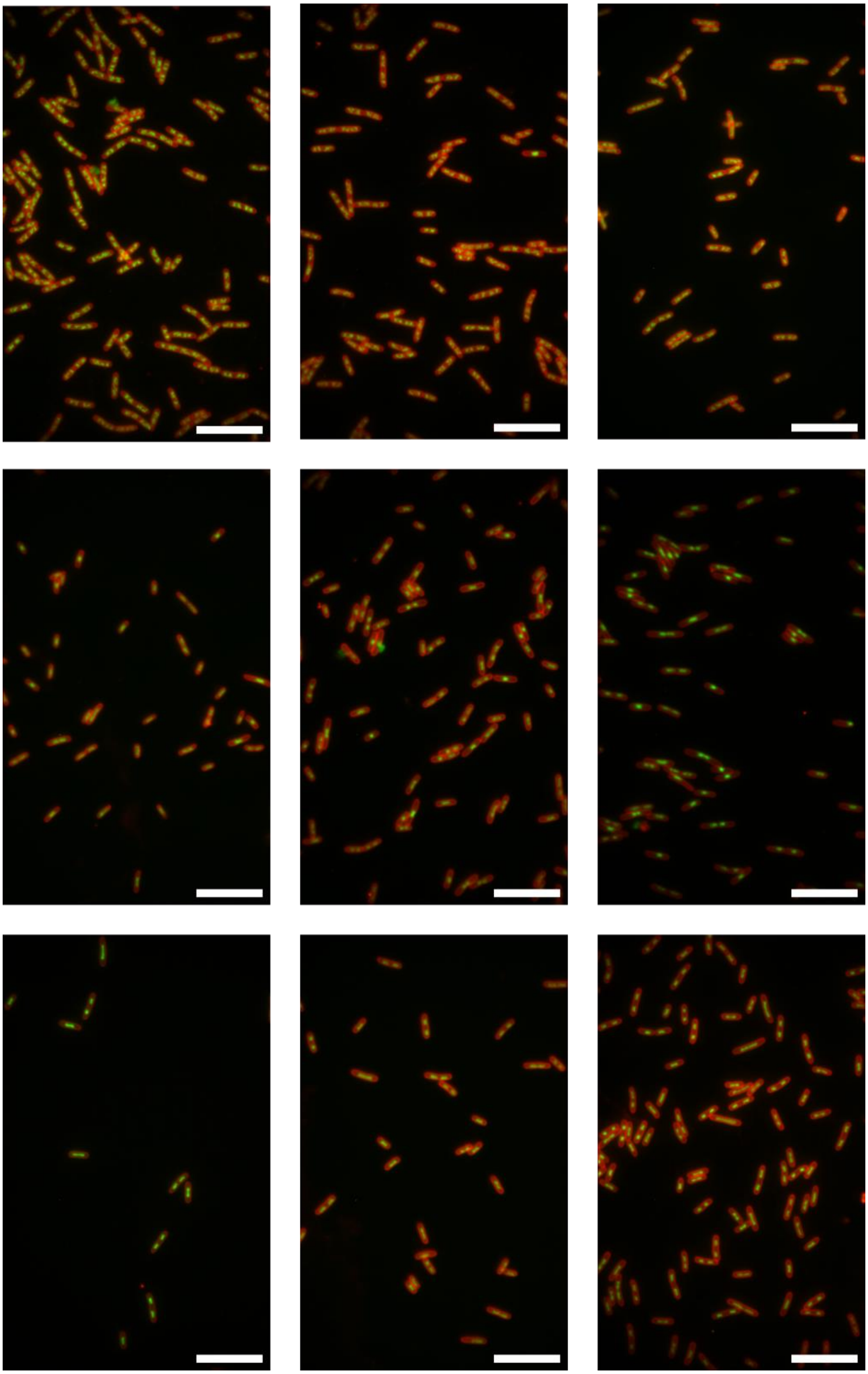

Fig S2. Representative fields of view containing MG1655 *E. coli* cells showing the untreated, ciprofloxacin and gentamicin susceptible phenotypes used for classifier training. (top row) 3 example fields of view showing the untreated phenotype, where no antibiotic was used. This phenotype was used to train the resistant class. (middle row) 3 example fields of view showing the ciprofloxacin susceptible phenotype, as a result of incubation with ciprofloxacin at concentration and duration shown in Fig S2. (bottom row) As above, but showing the gentamicin susceptible phenotype, the result of incubation with gentamicin. The scalebars are 5 µm.

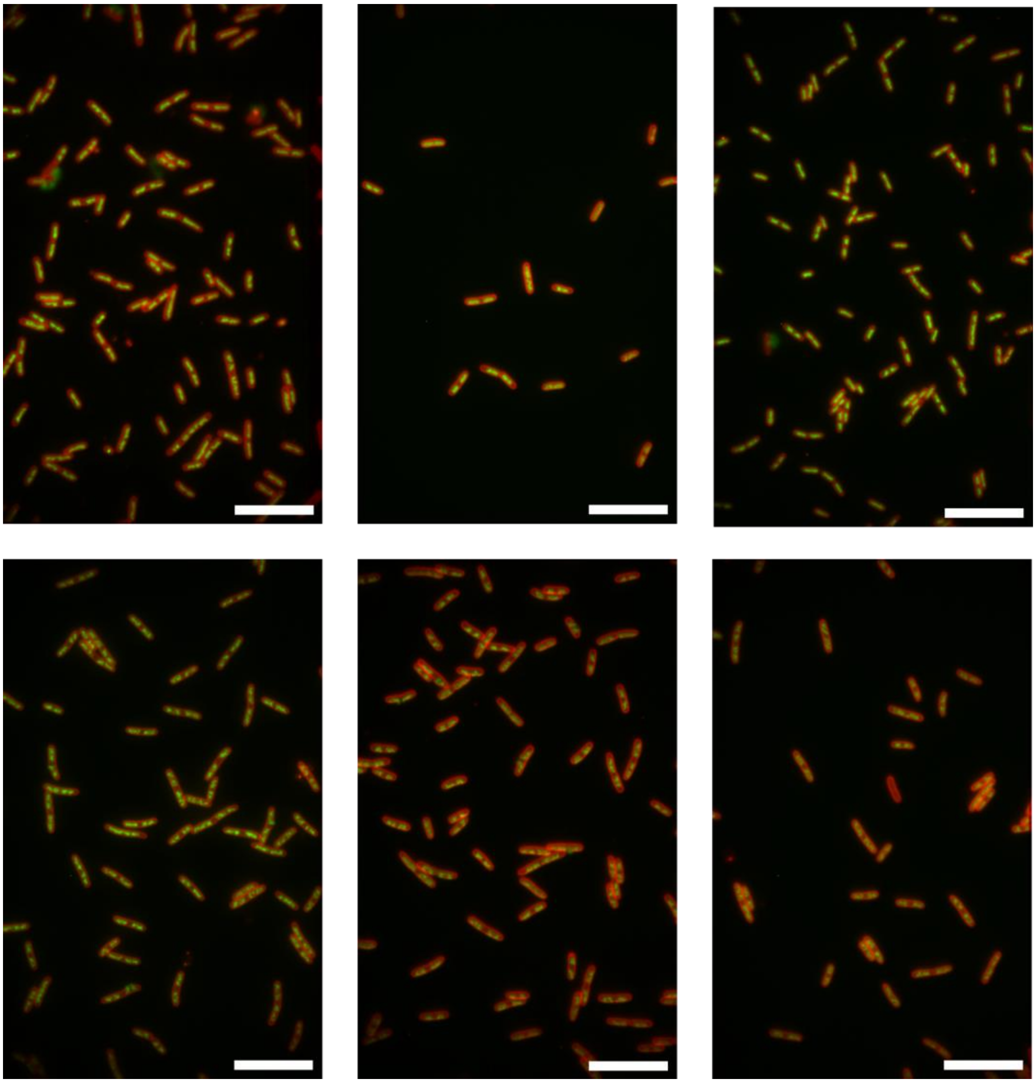

Fig S3. Representative fields of view containing MG1655 *E.coli* cells showing the rifampicin and co-amoxiclav susceptible phenotypes used for classifier training. (top row) 3 example fields of view showing the rifampicin susceptible phenotype, as a result of incubation with rifampicin at concentration and duration shown in Fig S2. (bottom row) As above, but showing the co-amoxiclav susceptible phenotype, the result of incubation with co-amoxiclav. The scalebars are 5 µm.

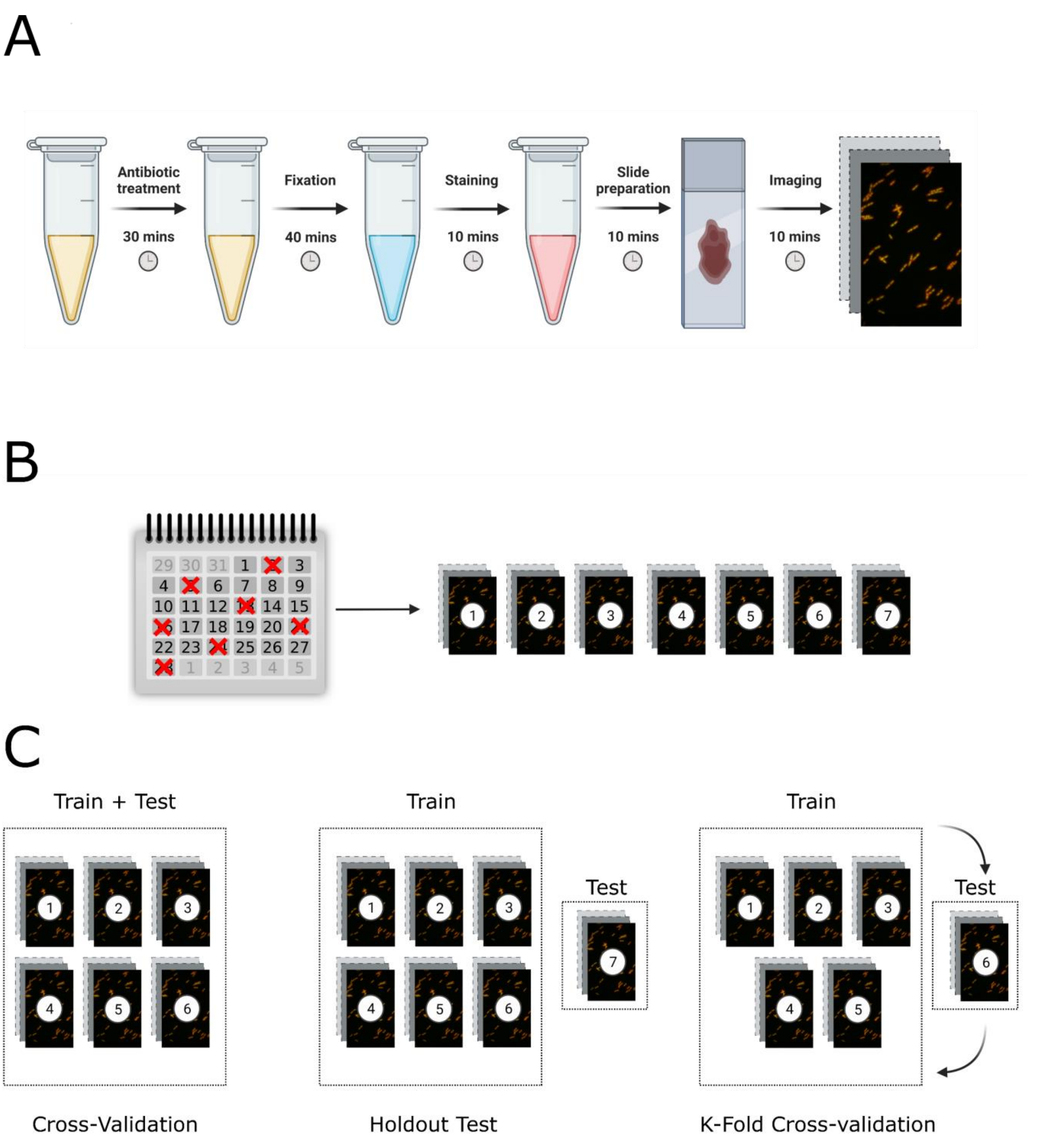

Fig S4. Experimental and computational setup. (A) Experimental setup. Live cells are treated with an antibiotic of choice, fixed with formaldehyde and permeabilised with ethanol. Fixed cells are stained with DAPI and Nile Red to stain the nucleoid and cell membrane respectively. The solution is placed on an agarose pad slide and imaged on an epifluorescence microscope. (B) 7 independent repeat experiments were conducted on 7 different days. 6 were used for training and cross-validation, one was the independent hold-out test. (C) Computational setup. (left) In the cross-validation experiment, all non-edge cells from 6 different independent experiments are aggregated together, and divided into training and testing sets. (middle) In the holdout test experiment, the same number of cells per class per experiment was selected randomly from each of the 6 experiments, and used to train the model. The model was tested the same number of cells per class from a 7th repeat experiment – this experiment was not used for pipeline optimization or hyperparameter tuning. (right) In the K-fold cross-validation experiment, the same number of cells per class per experiment were randomly selected from each of the 6 experiments. One experiment was withheld to be used as the test set, and remaining 5 were used for training. The test experiment was rotated 6 times – the final result is the sum over 6 different models, each trained and tested on a different permutation of repeat experiments.

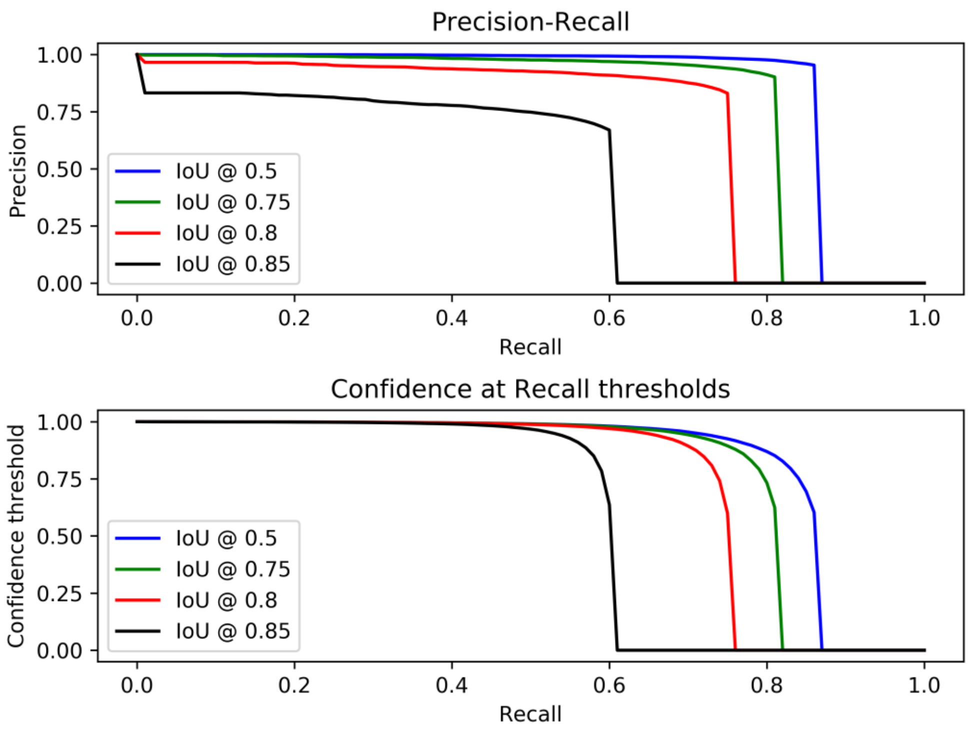

Fig S5. Precision-Recall and corresponding confidence across a range of intersection over union (IoU) thresholds of the Mask R-CNN segmenter, evaluated on all micrographs from the holdout experiment. Evaluated on 155 micrographs containing all of the phenotypes used in the study. (top) The precision-recall curve at 4 selected IoU thresholds. (bottom) The prediction confidence, corresponding to the precision-recall curve.

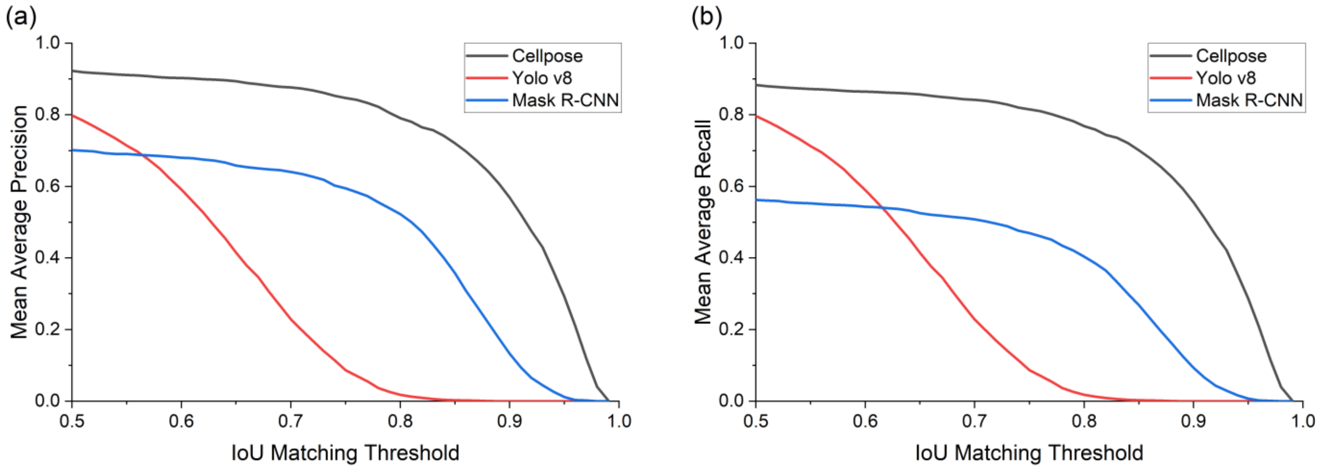

Fig S6. The mean average precision (a) and the mean average recall (b) for the segmentation models, Cellpose, Yolo v8 and Mask R-CNN. Evaluated on 155 micrographs containing all of the phenotypes used in the study.

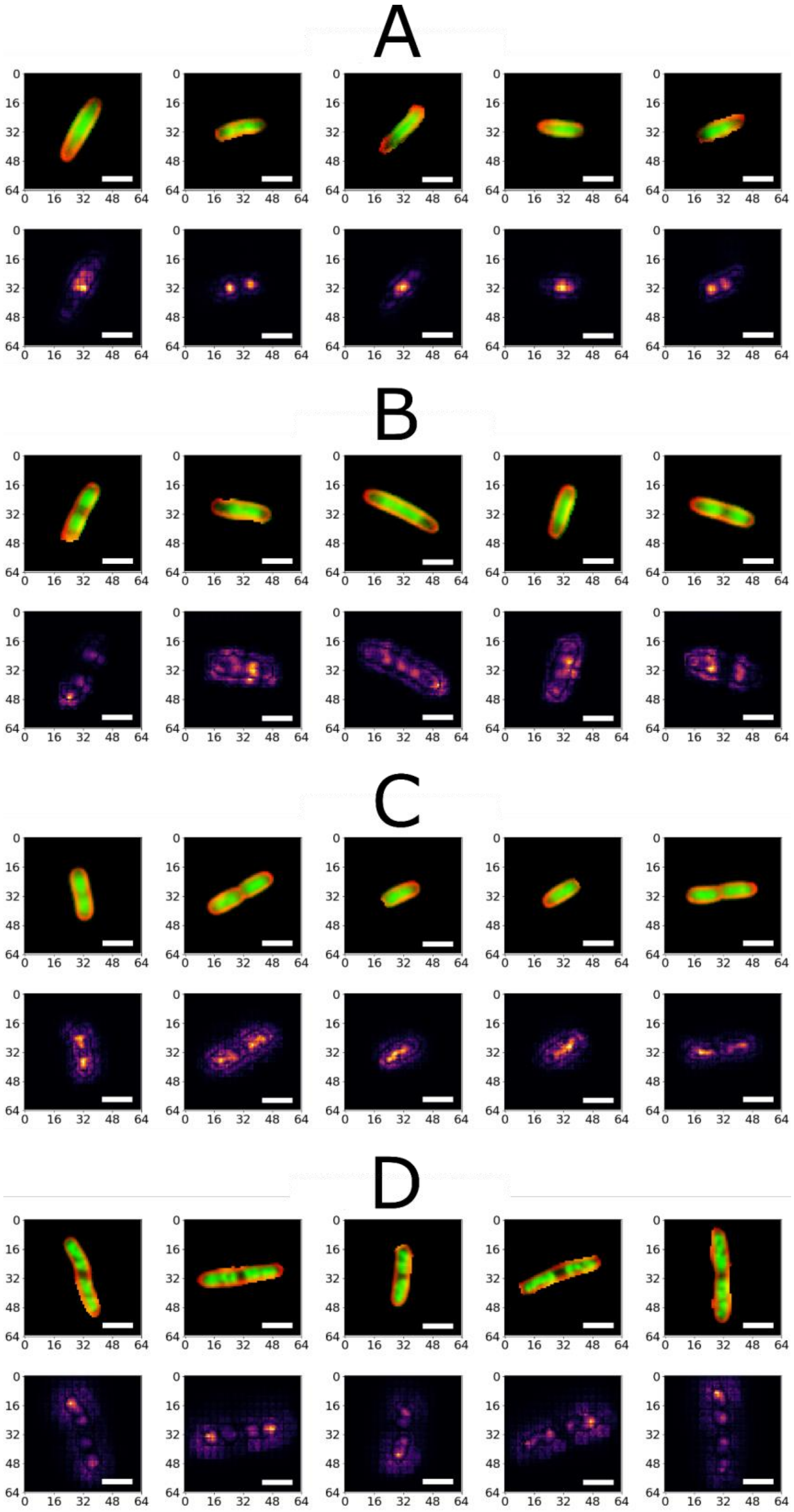

Fig S7. Saliency mapping of randomly selected, correctly classified susceptible cells. (A) (top) Randomly selected ciprofloxacin susceptible cells, after pre-processing steps, as presented to the classifier. All these cells were correctly classified as susceptible (bottom) Guided saliency maps corresponding to selected single-cell phenotypes, absolute value. Bright regions highlight areas that contributed most to the classification decision. (B) As above, but for gentamicin. (C) As above, but for rifampicin. (D) As above, but for co-amoxiclav. The scalebars are 5 µm.

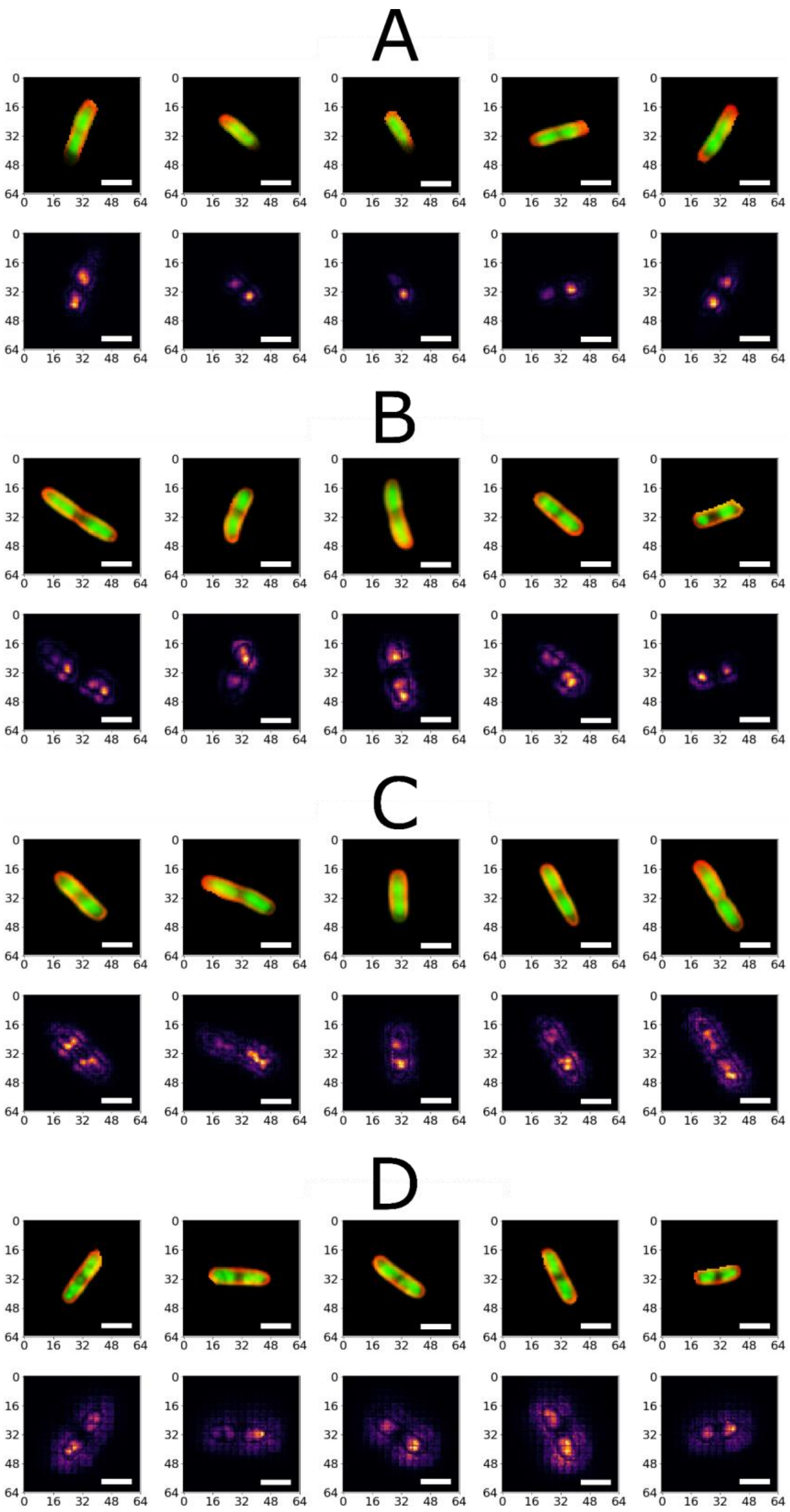

Fig S8. Saliency mapping of randomly selected, misclassified susceptible cells. (A) (top) Randomly selected, ciprofloxacin treated susceptible cells, after pre-processing steps, as presented to the classifier. All these cells were incorrectly classified as resistant. (bottom) Guided saliency maps corresponding to selected cells, absolute value. Bright spots highlight regions that contributed most to the classification decision. (B) As above, but for gentamicin. (C) As above, but for rifampicin. (D) As above, but for co-amoxiclav. The scalebars are 5 µm.

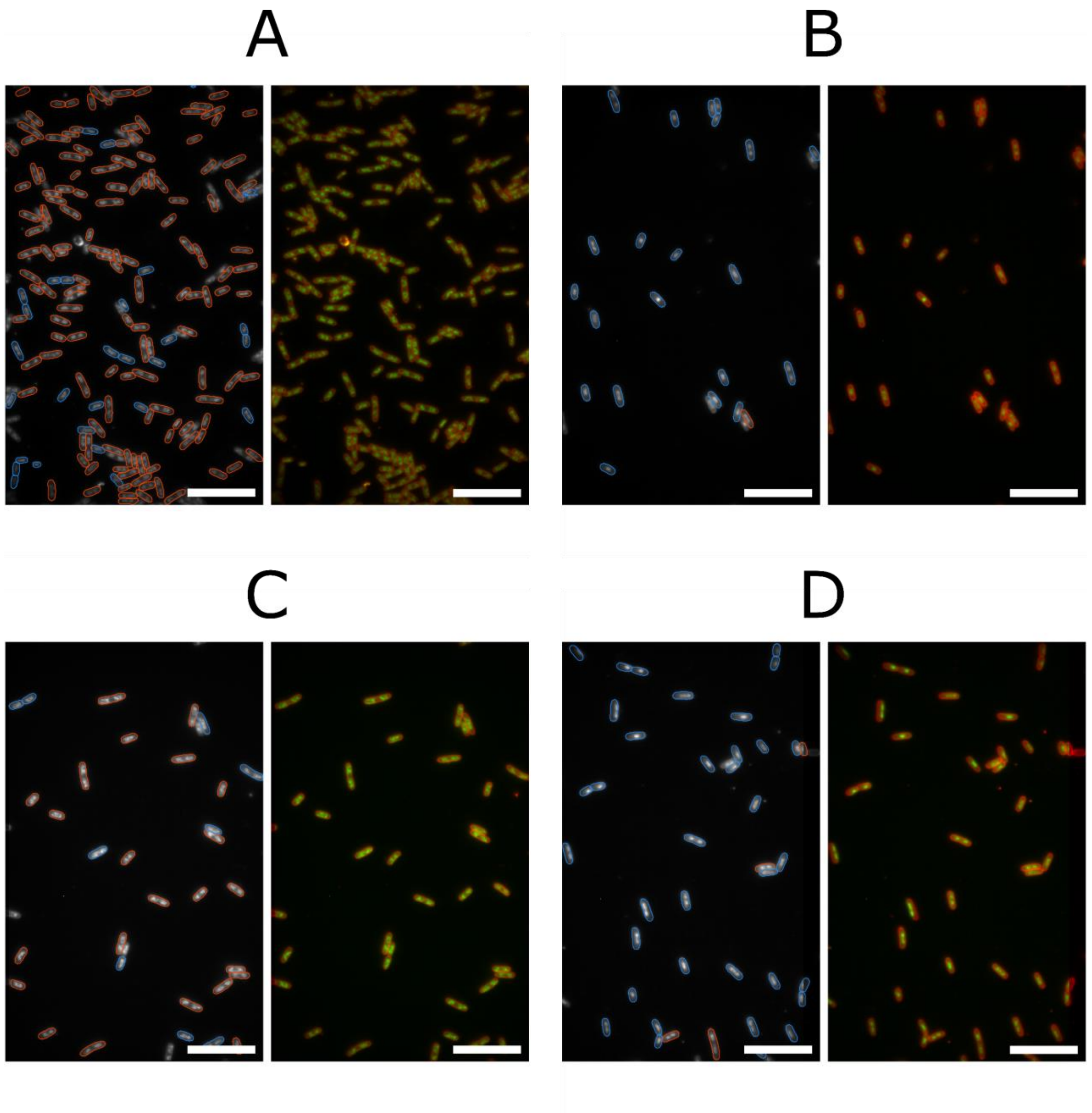

Fig S9. Representative fields of view and detection overlays of ciprofloxacin-susceptible clinical isolates EC1 and EC2, in both untreated and ciprofloxacin treated conditions. (A) (left) Detection overlay of a field of view of untreated EC2. Red detections are classified as resistant/untreated, blue detections are classified as susceptible. (right) Corresponding raw field of view. (B) As A, but for a ciprofloxacin treated EC2 field of view. (C) As A, but for untreated EC1. (D) As B, but for ciprofloxacin treated EC1. The scalebars are 5 µm.

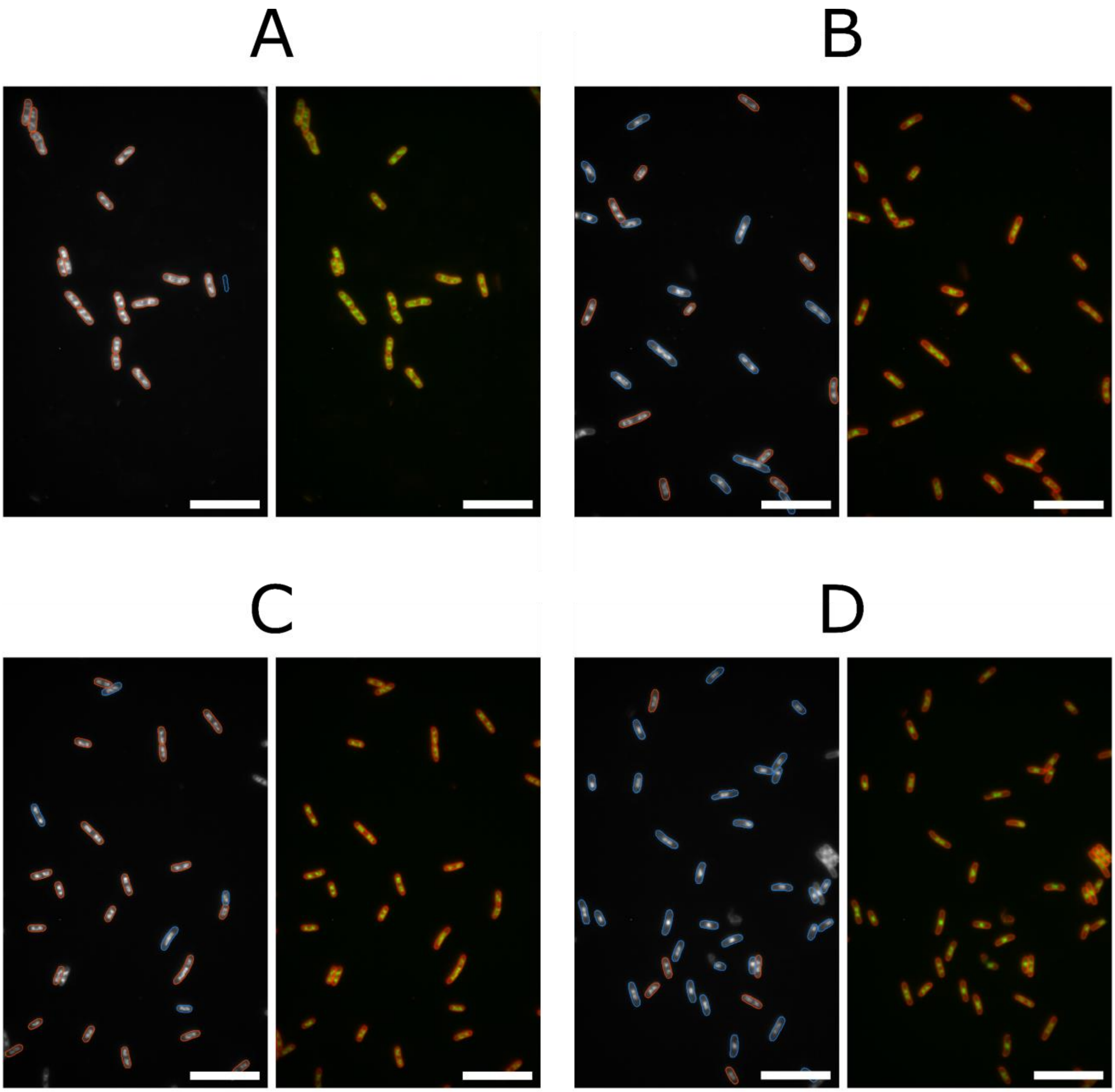

Fig S10. Representative fields of view and detection overlays of ciprofloxacin-susceptible clinical isolates EC3 and EC4, in both untreated and ciprofloxacin treated conditions. (A) (left) Detection overlay of a field of view of untreated EC4. Red detections are classified as resistant/untreated, blue detections are classified as susceptible. (right) Corresponding raw field of view. (B) As A, but for a ciprofloxacin treated EC4 field of view. (C) As A, but for untreated EC3. (D) As B, but for ciprofloxacin treated EC3. The scalebars are 5 μm.

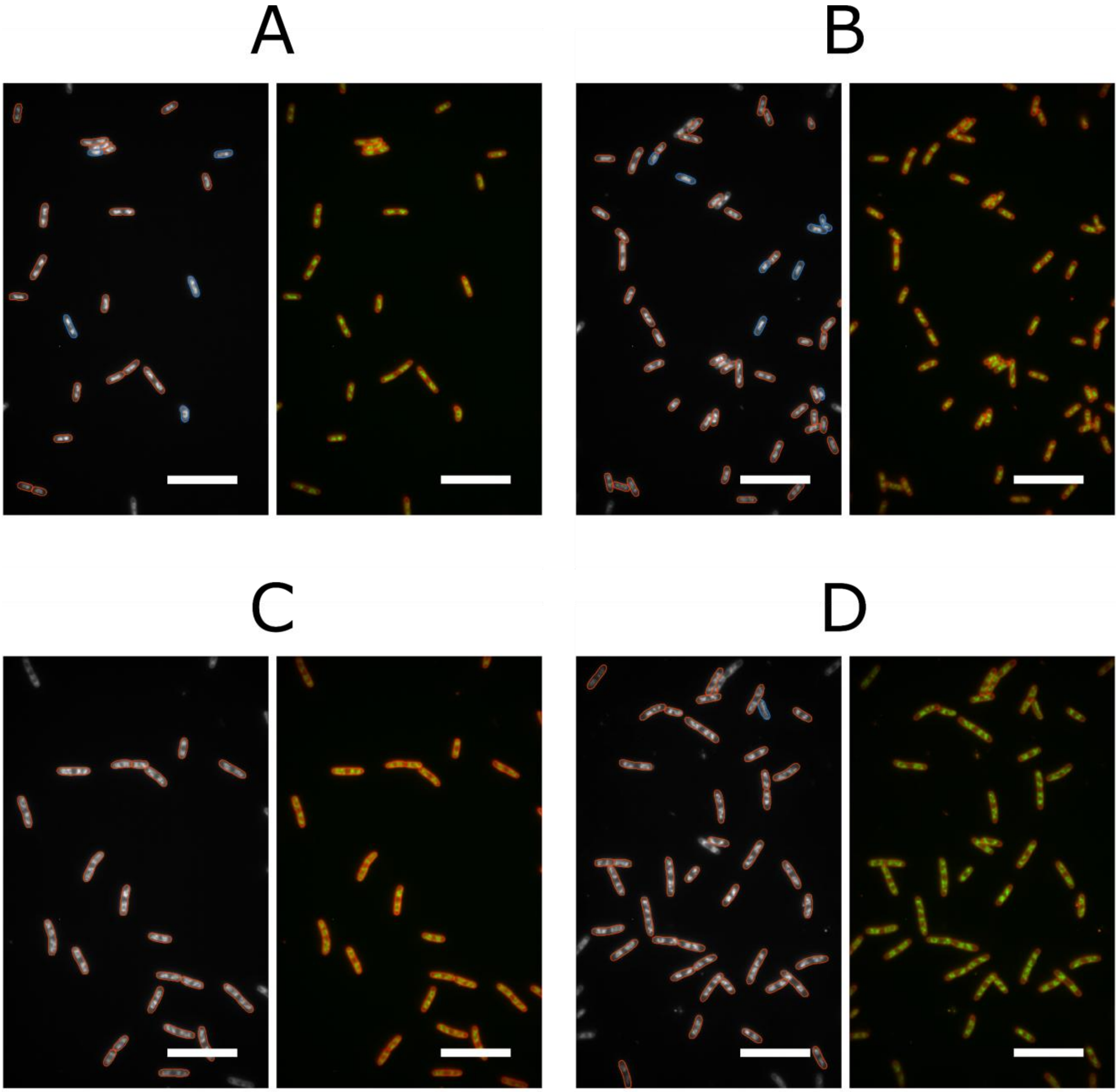

Fig S11. Representative fields of view and detection overlays of ciprofloxacin-resistant clinical isolates EC5 and EC6, in both untreated and ciprofloxacin treated conditions. (A) (left) Detection overlay of a field of view of untreated EC6. Red detections are classified as resistant, blue detections are classified as susceptible. (right) Corresponding raw field of view. (B) As A, but for a ciprofloxacin treated EC6 field of view. (C) As A, but for untreated EC5. (D) As B, but for ciprofloxacin treated EC5. The scalebars are 5 µm.

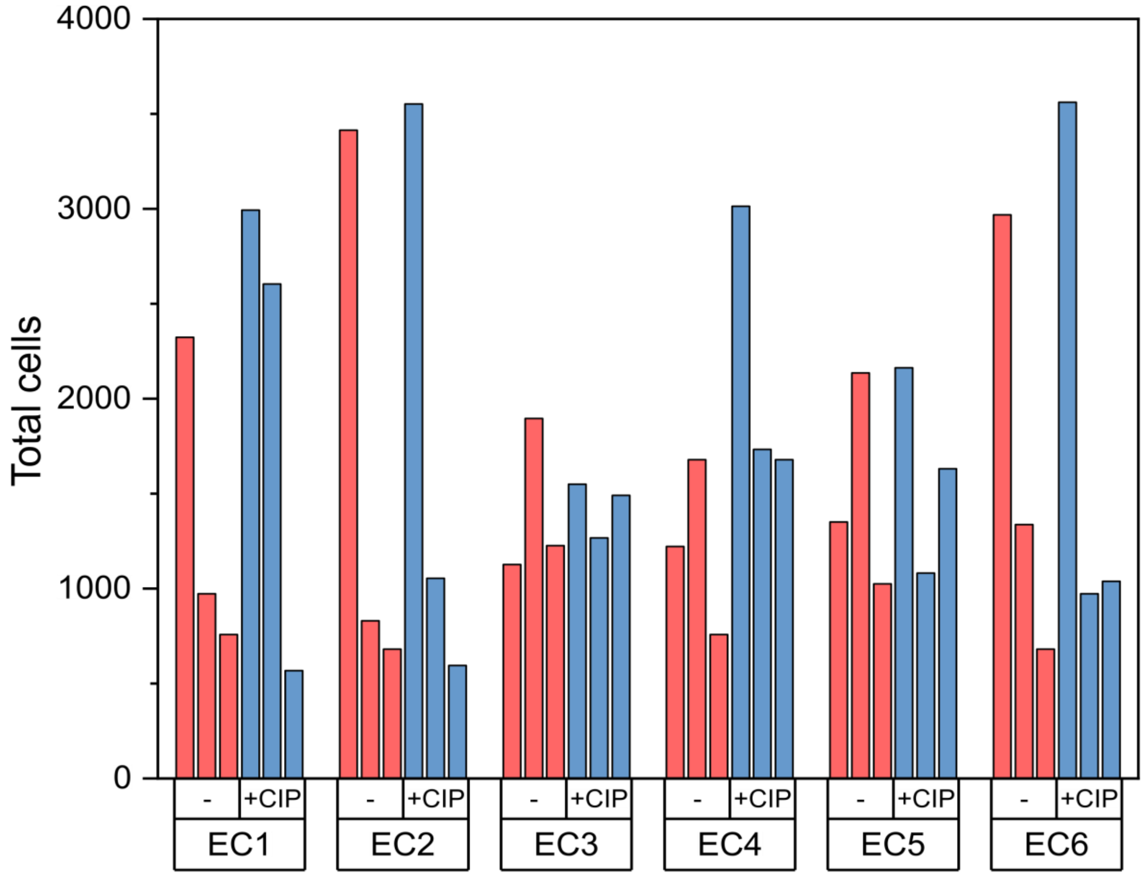

Fig S12. Total numbers of cells detected in each of the biological replicate experiments used to generate Figure 5, in both untreated and ciprofloxacin treated conditions. (top axis) Treatment condition. (bottom axis) Isolate code.

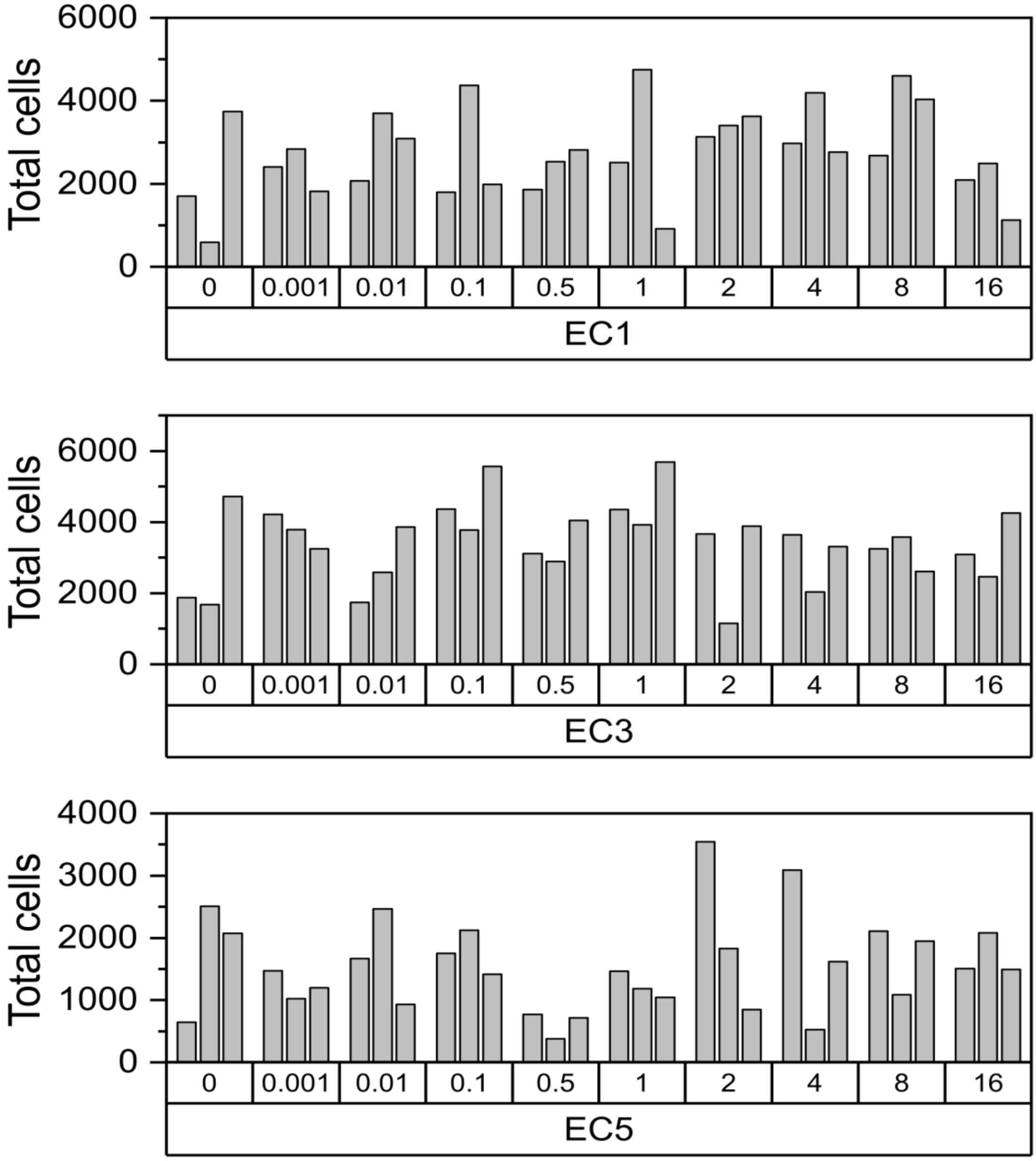

Fig S13. Total numbers of cells detected in each of the biological replicate experiments used to generate Figure 6, in both untreated and ciprofloxacin treated conditions. (top axis) Ciprofloxacin treatment concentration in mg/L. “0” indicates no antibiotic treatment. (bottom axis) Isolate code.

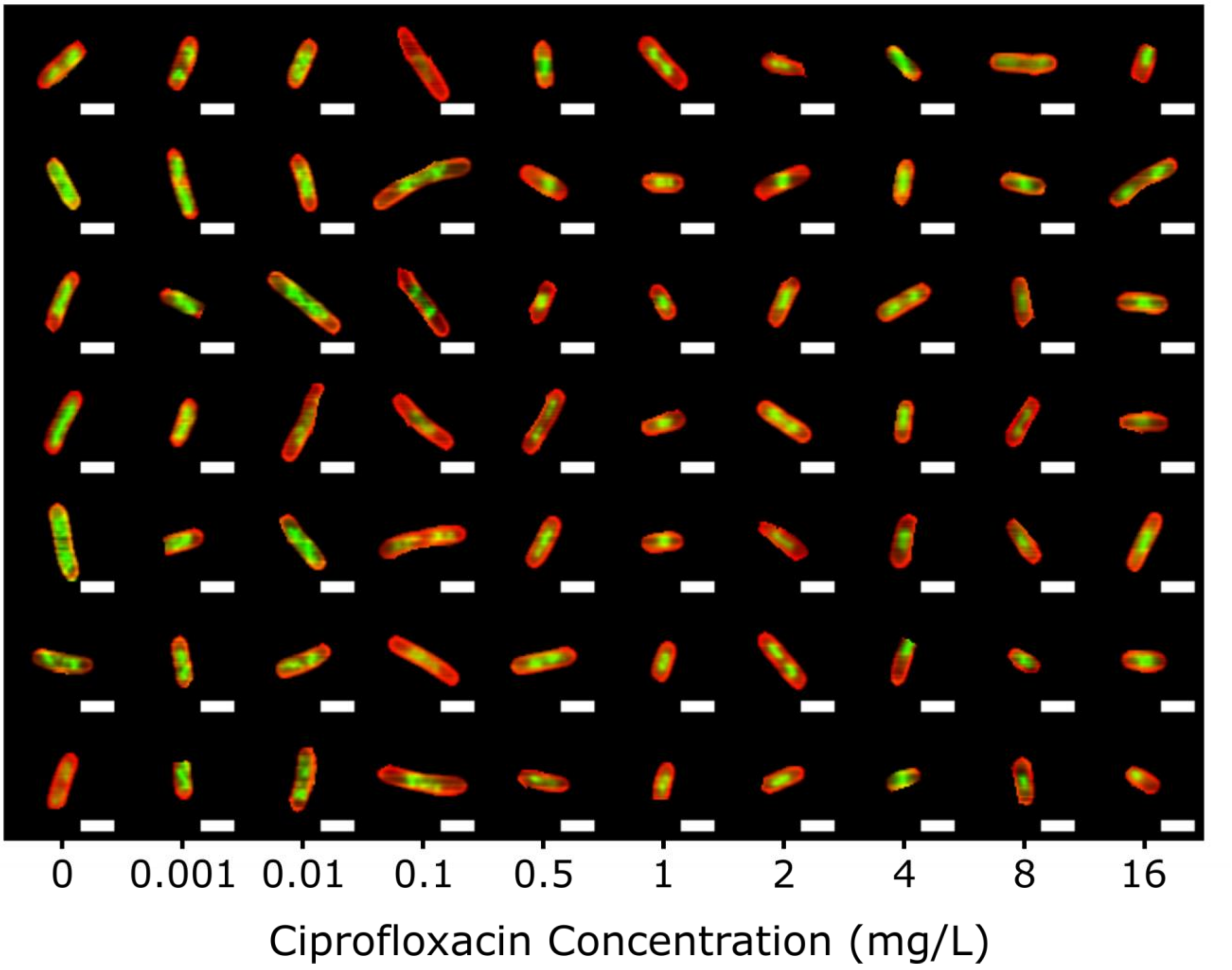

Fig S14. Example images from the titration dataset shown in figure 6, for the clinical isolate EC1. The measured MIC for the clinical isolate EC1 is 0.008 mg/L.

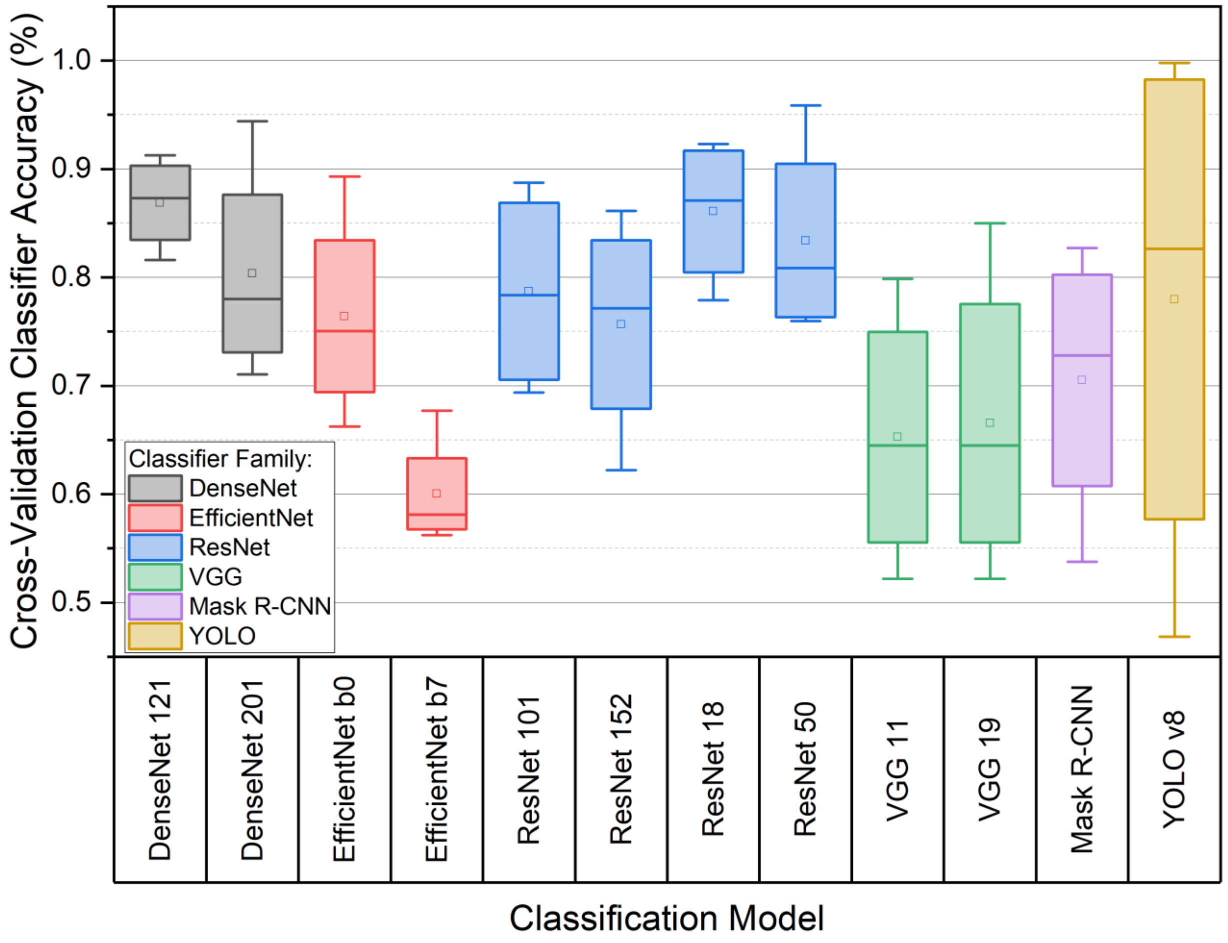

Fig S15. Box plots of classification accuracy for a range of different convolutional neural network architectures, evaluated over the four antibiotic phenotypes on the cross-validation test set. The learning rate and batch size were found for each neural network using a grid search. Densenet121 was found to have both the highest mean accuracy, and the most consistent performance across all antibiotic phenotypes.

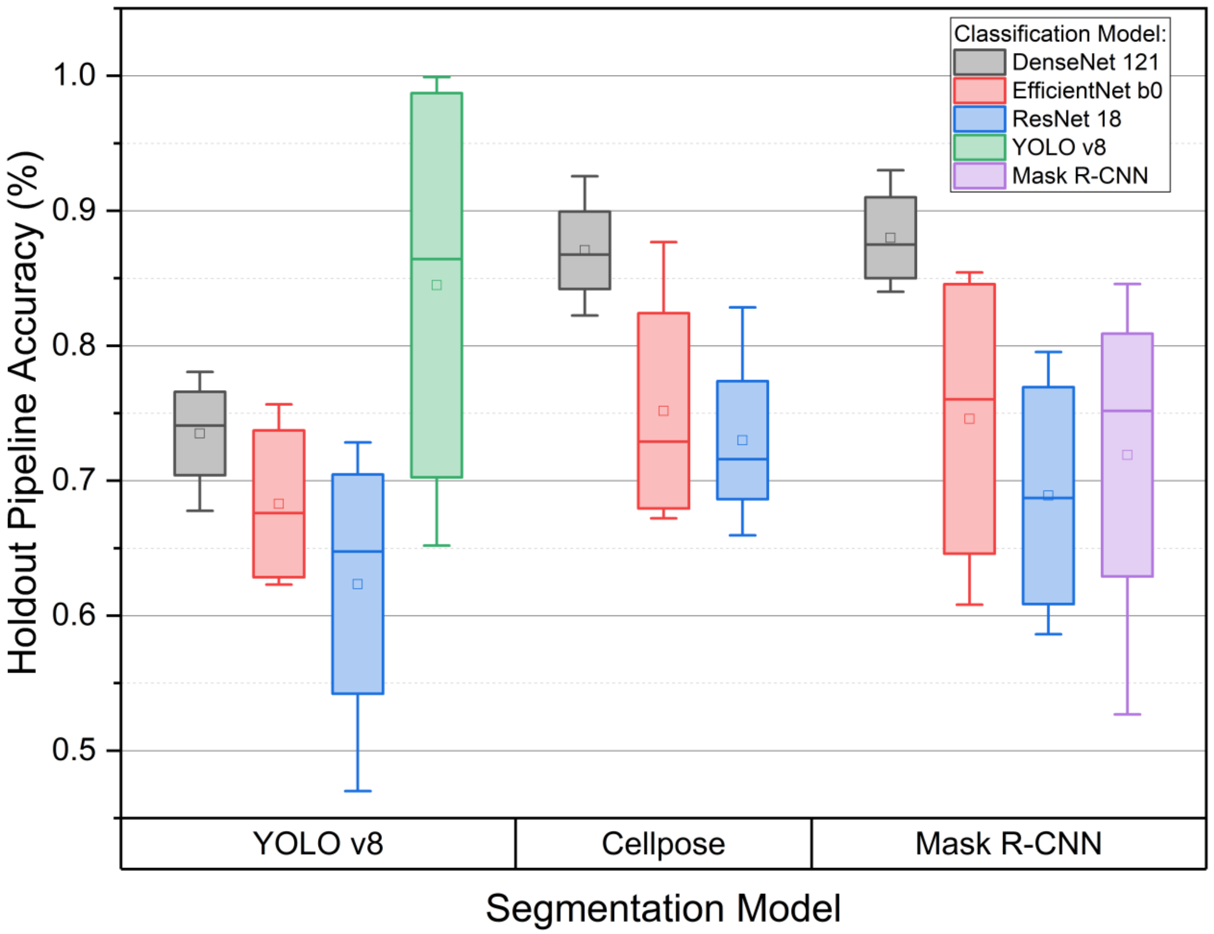

Fig S16. Box plots of the pipeline classification accuracy while utilising different segmentation models and classification models, evaluated over the four antibiotic phenotypes on the holdout test set. The learning rate and batch size were found for each neural network using a grid search. A pipeline featuring Mask R-CNN + Densenet121 was found to have the highest mean accuracy across all antibiotic phenotypes.

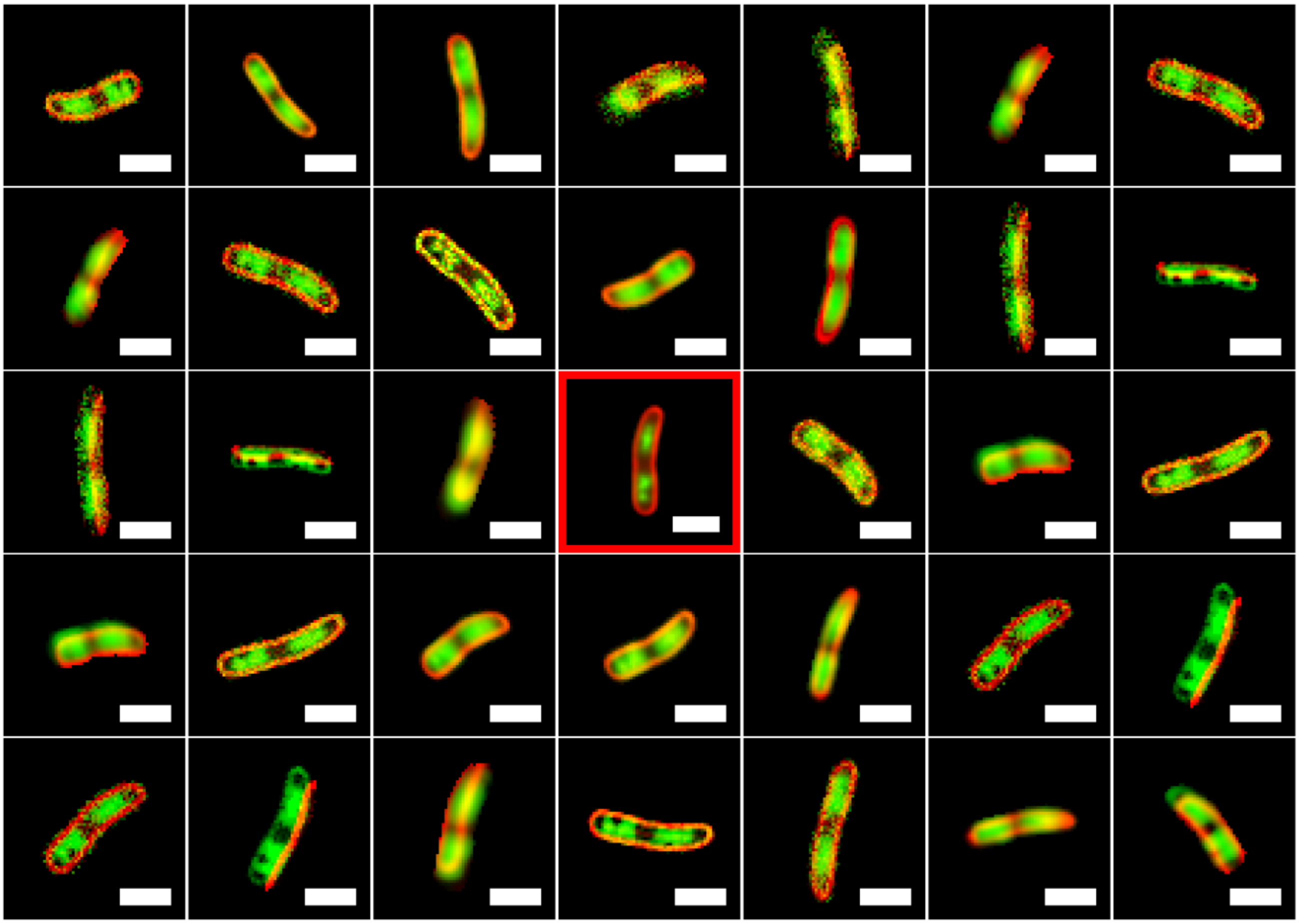

Fig S17. Example image augmentations that are applied during training. The centre image (highlighted in red) is unperturbed, whereas every other image has been randomly augmented using a random sequence of transformations including horizontal and vertical flips and translations, rotations, cutout as well as Gaussian blurring. The scalebar is 2 µm.

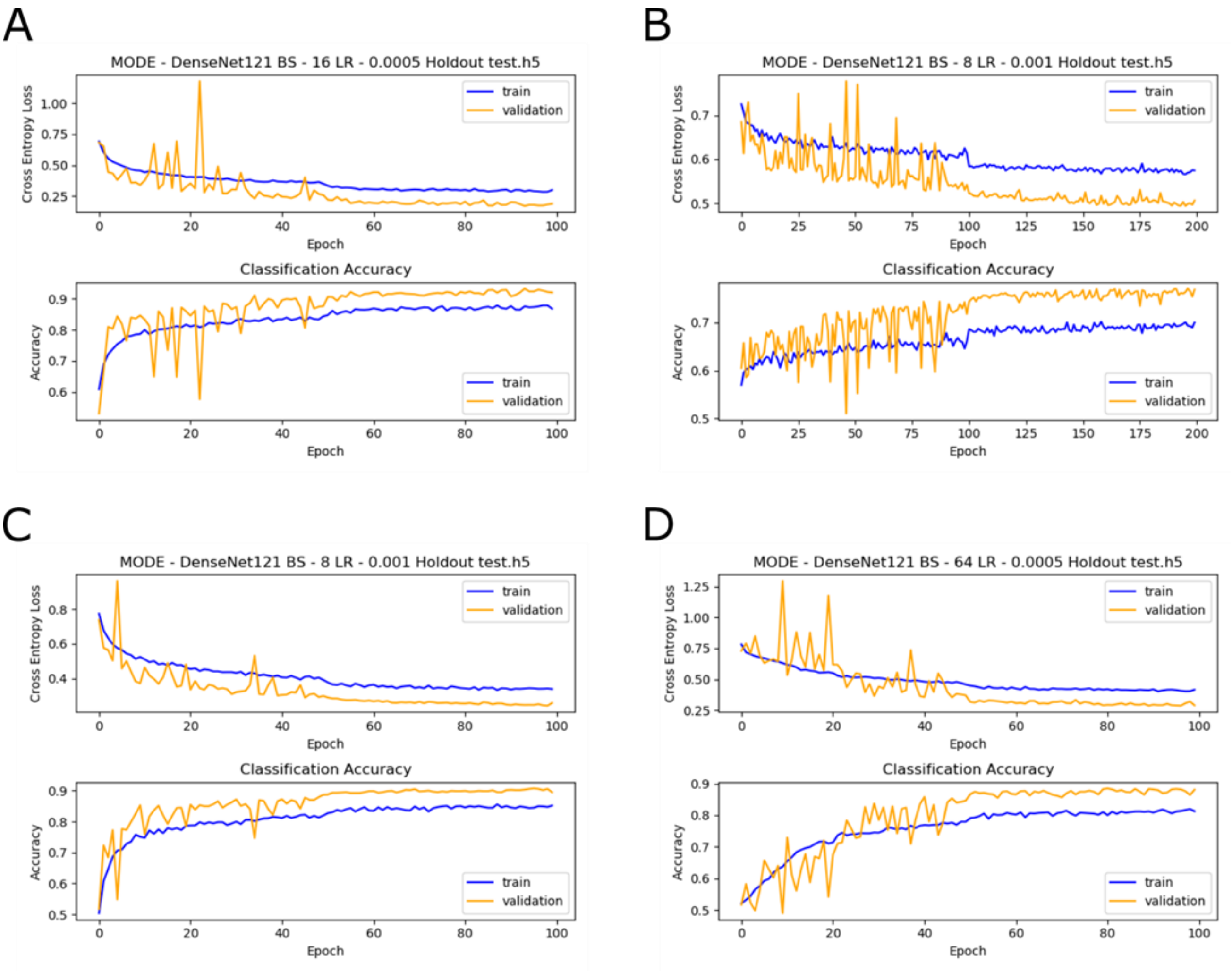

Fig S18. DenseNet 121 training loss and training accuracy logs, evaluated on the train and validation datasets, for (A) Ciprofloxacin, (B) Co-amoxiclav, (C) Gentamicin, (D) Rifampicin. To prevent overfitting the train dataset is randomly augmented during training, whereas the validation dataset it not augmented.
