# Supplementary material for: Deep learning and single-cell phenotyping for rapid antimicrobial susceptibility detection in Escherichia coli
Source: Commun Biol. 2023 Nov 14;6:1164. doi: 10.1038/s42003-023-05524-4 (PMC10645916; doi:10.1038/s42003-023-05524-4)
Supplement: Supplementary file 3 — Description of Additional Supplementary Files [file 42003_2023_5524_MOESM3_ESM.pdf]

Description of Additional Supplementary Files

File name: Supplementary Dataset 1
Description: Source data for figure 5

File name: Supplementary Dataset 2
Description: Source data for figure 6

File name: Supplementary Dataset 3
Description: Source data for figure S15

File name: Supplementary Dataset 4
Description: Source data for figure S16
